# Activation of RAAS in a rat model of liver cirrhosis: no effect of losartan on renal sodium excretion

**DOI:** 10.1186/s12882-018-1039-6

**Published:** 2018-09-19

**Authors:** A. D. Fialla, O. B. Schaffalitzky de Muckadell, P. Bie, H. C. Thiesson

**Affiliations:** 1 Department of Gastroenterology and Hepatology, Odense University Hospital, Sdr Boulevard, 5000 Odense C 29 Odense, Denmark; 2 Department of Nephrology, Odense University Hospital, Odense, Denmark; 3 Cardiovascular and Renal Research, University of Southern Denmark, Odense, Denmark

**Keywords:** Sodium retention, Bile duct ligation, ANGII antagonists, Liver cirrhosis, Aldosterone

## Abstract

**Background:**

Liver cirrhosis is characterized by avid sodium retention where the activation of the renin angiotensin aldosterone system (RAAS) is considered to be the hallmark of the sodium retaining mechanisms. The direct effect of angiotensin II (ANGII) on the AT-1 receptor in the proximal tubules is partly responsible for the sodium retention. The aim was to estimate the natriuretic and neurohumoral effects of an ANGII receptor antagonist (losartan) in the late phase of the disease in a rat model of liver cirrhosis.

**Methods:**

Bile duct ligated (BDL) and sham operated rats received 2 weeks of treatment with losartan 4 mg/kg/day or placebo, given by gastric gavage 5 weeks after surgery. Daily sodium and potassium intakes and renal excretions were measured.

**Results:**

The renal sodium excretion decreased in the BDL animals and this was not affected by losartan treatment. At baseline the plasma renin concentration (PRC) was similar in sham and BDL animals, but increased urinary excretion of ANGII and an increase P-Aldosterone was observed in the placebo treated BDL animals. The PRC was more than 150 times higher in the losartan treated BDL animals (*p* < 0.001) which indicated hemodynamic impairment.

**Conclusions:**

Losartan 4 mg/kg/day did not increase renal sodium excretion in this model of liver cirrhosis, although the urinary ANGII excretion was increased. The BDL animals tolerated Losartan poorly, and the treatment induced a 150 times higher PRC.

## Background

Liver cirrhosis is characterized by avid sodium retention, where several mechanisms are considered to be responsible. Impaired renal sodium handling is partly due to increased angiotensin II (ANGII) as well as aldosterone levels and its action at the proximal, distal and collecting tubules in the kidneys. Both ANGII and aldosterone are expected to be increased in cirrhosis secondary to the central vasodilation, reduced effective blood volume, where the neurohumoral response maintain blood pressure [1]. ANGII exerts its action mainly through the AT-1 receptor and leads to constriction of the renal artery and increases sodium reabsorption in the proximal tubule [2]. Secondly, ANGII promotes aldosterone release from the adrenal gland which in concert with ANGII regulates the hydro-electrolytic balance [3]. The action of an AT1 receptor antagonist (losartan) is anticipated to be an increased renal sodium excretion and reduced aldosterone release. The bile duct ligated (BDL) rat model of cirrhosis is characterized by portal hypertension and sodium retention [4]. The activity of RAAS has in this model been found contradictory; normal renin coexisting with either low or high levels of aldosterone [4, 5] or coexisting increased levels of renin, ANGII and aldosterone [6]. One study found increased levels of intrarenal components of RAAS irrespective of the systemic RAAS [7]. Although systemic RAAS activation does not fully explain the sodium retention in the BDL model, the sodium excreting effects of losartan, an ANGII receptor antagonist, has previously been demonstrated where long term treatment in the early course of disease did not increase sodium excretion [8] whereas low doses in the late phase given short term increased sodium excretion [9].

Yet has to be explored, whether long-term treatment with ANGII receptor antagonists may improve sodium excretion in the late phase of the diseases where formation of ascites is frequent. It is hypothesized, that long-term treatment with losartan increases sodium excretion in the BDL model of cirrhosis in the late course of disease by direct blockage of the AT1 receptor and reduction of aldosterone secretion.

## Methods

### Animals and surgical protocol

The experimental model used is the common bile duct ligated rat (BDL) [10, 11]. Male Wistar rats (17 weeks) were obtained from M&B /Ejby, Denmark. The animals where randomized to BDL or sham surgery., and the group allocation was blinded to the investigator. They were given a 0.2% sodium and 1.0% potassium diet (Altromin 1324;Altromin International, Lage, Germany) and had free access to tap water. Food was administered as granules in metabolic cages to avoid spillage into the urine collection vials. For surgery the animals were anesthetized by subcutaneous injection of fentanyl citrate (0.25 mg/kg), fluanosone (8 mg/kg), and diazepam (4 mg/kg). In BDL rats the common bile duct was isolated, ligated and 0.5 cm was excised. In sham rats the common bile duct was isolated, manipulated and left intact. Postoperative pain was treated with buprenorphin subcutaneously (0.1 mg/kg). At the end of the treatment protocols the rats were anaesthetized by CO_2_ inhalation shortly before decapitation and trunk blood was collected for analysis. Organs were removed and weighed. Ascites volume was estimated by aspiration of free fluid from the abdominal cavity. In two BDL rats invasive blood pressure measurement was performed. The Danish Animal Experiments Inspectorate approved all experimental procedures and all animals were treated according to the “Guide for the Care and Use of Laboratory Animals”.

### The experimental design

**Fig. 1:**
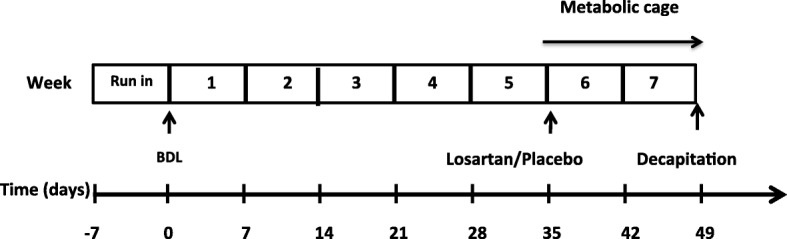
Study design

Four groups consisting of two BDL groups (*n* = 20) and two sham groups (*n* = 16) were followed for 34 days after surgery after which they were placed in individual metabolic cages. Day 35, baseline measures were obtained and treatment initialized on day 36. The animals were treated with a daily dose of losartan (Cozaar©, MSD, Denmark; 4 mg/kg) or placebo (tap water) for 14 days (Fig. 1).The treatment was blinded to the investigator of the results. The treatment was administered by gastric gavage twice a day. Na^+^, K^+^, water intake and excretion and ANGII excretion were measured daily. Animals that did not complete the 2 week 24 h collections were excluded from the data analysis.

### Plasma and urine analyses

*Hormones*: Plasma renin concentration (PRC) was measured by radioimmunoassay as previously described [12]. ANGII in urine was determined using a specific antibody (Ab-5-030682) as previously described [13]. Plasma aldosterone was measured with a commercial kit (Coat-A-Count Aldosterone, DPC, Los Angeles). *Biochemistry*: S-Bilirubin, S-Albumin, S-creatinine were analyzed using Cobas Mira Plus analyzer (Roche Diagnostics, Basel). Platelet count, leucocytes, hematocrit and hemoglobin were analyzed on a Celltacα MEK-6108 K (Nihon Kohden, Tokyo, Japan). *Electrolytes in urine and plasma* were determined by flame photometry (IL 943 flame photometer; Instrumentation Labatory, Milan, Italy) Osmolality was measured by freezing point depression (Osmomat 030D; Gonotec, Berlin, Germany). *Calculations****:*** Sodium and potassium excretion were calculated as the urinary excretion normalized to the body weight on a daily basis. The renal sodium and potassium retention was calculated as the intake minus urinary excretion. *Blood pressure measurement:* Blood pressure was measured with the TA11PA-C40 transmitter (Data Sciences International, St. Paul, Mn, USA), which was implanted in the abdominal aorta under general anesthesia one week before the start of data collection. In conscious animals measurements were recorded every minute for 24 h a day for 4 days a week in all 7 weeks. The systolic and diastolic blood pressures were recorded, whereas mean arterial pressure and heart rate were calculated. The multiple blood pressure recordings where reported as mean and SD at the end of week 1 and the end of week 7. Dataquest A.R.T software, version 2.10 was applied (Data Sciences International, St. Paul, Mn, USA).

### Statistical analysis

Water intake, food intake, urine volume, sodium and potassium intake, sodium and potassium excretion were all reported as summary measures for the first and second week after treatment initiation (week 6 + 7). The sodium and potassium excretion is given as the excretion/intake. Results are given as mean ± standard error of the mean (SEM). For group comparison the Kruskal-Wallis test with post test was applied. Calculations were performed Graph Pad/Prism 5.2, When comparing groups, the difference within the sham -operated animals as well as the BDL animal was analyzed as well as the difference between the BDL animals and their respective sham operated controls. Sample size calculation was not performed, since the experiment was initially explorative.

## Results

### Animal characteristics

The number of rats completing the protocol was 8 out of 10 in BDL + placebo group and 6 out 10 in the BDL + losartan group. In the BDL + placebo group 2 animal died on day 7 after treatment due to intraperitoneal bleeding. Both animals had ascites. In the BDL losartan group 1 animal was found dead on day 6 due to intraperitoneal bleeding. 3 animals where terminated on day 5, 10 and 14 according to the rules of animal welfare. One animal had ascites. All the sham operated rats completed the protocol. Preoperative weight was similar in all four groups. The final ascites free weight (dry) was significantly reduced in the BDL animals compared to their respective controls. Ascites developed in 7/8 rats in the BDL+ placebo group and in 3/6 animals in the BDL + losartan group. In the sham operated animals the spleen, the liver, the kidneys and the heart were all smaller than in the BDL animals. The spleen was significantly smaller in the losartan treated BDL animals compared to the placebo treated BDL animals. S-bilirubin and B-leucocytes were significantly higher in the BDL animals and S-albumin as well as the platelet count was lower compared to the sham controls. P-potassium was higher in the losartan treated BDL animals compared to the placebo treated BDL animals (Table 1).

**Table 1:** Demografic and biochemical data

	Unit	Sham + placebo	Sham + losartan	BDL + placebo	BDL + losartan
Initial number rats	N	8	8	10	10
Mortality	N	0	0	3	6
Rats with/ without ascites	N	0\8	0\8	7\1	3\3
Preoperative weight	g	237 ± 5	243 ± 9	240 ± 4	237 ± 7
Final weight	g	346 ± 14	366 ± 15	325 ± 5	317 ± 9
Final weight (dry)	g	346 ± 14	366 ± 15	317 ± 6	314 ± 8
Weight gain (dry)	g	109 ± 13^†^	123 ± 12^†^	77 ± 5	77 ± 8
Liver	(g/100gBW)	3.37 ± 0.17^†††^	3.31 ± 0.05^†††^	7.41 ± 0.37	7.09 ± 0.22
Spleen	(g/100gBW)	0.19 ± 0.01^†††^	0.19 ± 0.004^†††^	0.62 ± 0.02	0.50 ± 0.05*
Kidney	(g/100gBW)	0.56 ± 0.08^††^	0.60 ± 0.01^†^	0.76 ± 0.01	0.80 ± 0.03
Heart	(g/100GBW)	0.27 ± 0.01^†^	0.25 ± 0.01^††^	0.33 ± 0.02	0.28 ± 0.02
Adrenal gland	(g/100gBW)	0.016 ± 0.001	0.015 ± 0.001	0.018 ± 0.001	0.018 ± 0.001
B-Haemoglobin	mmol/L	9.7 ± 0.1	9.7 ± 0.1	9.6 ± 0.4	9.9 ± 0.6
B-Haematocrit	%	47 ± 1	46 ± 1	49 ± 1	49 ± 3
B-Leucocytes	10*9/L	5.8 ± 0.4^†††^	6.3 ± 0.3^†††^	26.0 ± 2.3	23.9 ± 4.7
B-Platelets	10*9/L	791 ± 33	769 ± 41	567 ± 113	597 ± 56
S-Bilirubin	μmol/l	1.3 ± 0.16^†††^	0.8 ± 0.17^†††^	188.6 ± 10.8	227.3 ± 18.6
S-Albumin	μmol/l	667.3 ± 35.7^†††^	607.6 ± 23.1^†^	475.0 ± 25.2	514.8 ± 18.4
S-Creatinine	μmol/l	17.6 ± 0.6	16.3 ± 2.1	18.3 ± 1.3	21.5 ± 2.6
P-Osmolality	mOsm/kg	305 ± 2	306 ± 2	300 ± 1	304 ± 2
P-Sodium	mmol/l	139 ± 1.0	139 ± 0.8	141 ± 0.4	140 ± 0.9
P-Potassium	mmol/l	6.3 ± 0.2	6.5 ± 0.4	5.8 ± 0.2	7.1 ± 0.5*

Values are given as mean ± SEM. *p < 0.05: BDL placebo vs. BDL losartan. ^†^*p* < 0.05: Sham vs. BDL, ^††^*p* < 0.01 Sham vs. BDL, ^†††^*p* < 0.001: Sham vs. BDL

### Food and water intake

The sham-operated animals ate significantly more than the BDL animals*.* This was consistent with higher intake of sodium and potassium in the sham animals compared to the BDL animals. Water intake was similar in all four groups (Table 2).

**Table 2:** Water, food,electrolyte intake and electrolyte excretion in sham and BDL animals

	Unit		Sham + placebo	Sham + losartan	BDL + placebo	BDL + losartan
Water intake	ml/100 g BW*24 h	Week 6	9.8 ± 0.2	9.9 ± 0.1^†^	9.7 ± 0.3	9.9 ± 0.5
		Week 7	9.7 ± 0.2	9.9 ± 0.2^†^	9.2 ± 0.3	10.6 ± 0.6
Food intake	μg/100 g BW*24 h	Week 6	21.4 ± 0.3^†††^	21.6 ± 0.4^†††^	16.8 ± 0.6	17.4 ± 0.5
		Week 7	20.8 ± 0.3^†††^	21.1 ± 0.3^†††^	15.3 ± 0.4	15.7 ± 0.5
Sodium intake	μmolNa/100 g BW*24 h	Week 6	586 ± 9^†††^	580 ± 9^†††^	483 ± 17	504 ± 12
		Week 7	554 ± 9^†††^	553 ± 7^†††^	430 ± 11	459 ± 10
Potassium intake	μmolK/100 g BW*24 h	Week 6	1697 ± 25^†††^	1679 ± 27^†††^	1398 ± 49	1460 ± 35
		Week 7	1604 ± 27^†††^	1599 ± 21^†††^	1243 ± 32	1327 ± 46
Diureses	ml/100 g BW*24 h	Week 6	5.0 ± 0.1	5.1 ± 0.1	5.8 ± 0.2	5.8 ± 0.4
		Week 7	5.2 ± 0.2	5.3 ± 0.2	5.7 ± 0.3	6.8 ± 0.5
Osmolality	mOsm/kg	Week 6	1.50 ± 0.03	1.52 ± 0.04	1.36 ± 0.06	1.42 ± 0.06
		Week 7	1.39 ± 0.04^†††^	1.41 ± 0.04^†††^	1.21 ± 0.06	1.08 ± 0.05

Values given as mean ± sem, ^††^*p* < 0.01 Sham vs. BDL, ^†††^*p* < 0.001: Sham vs. BDL

### Sodium and potassium excretion

**Fig. 2:**
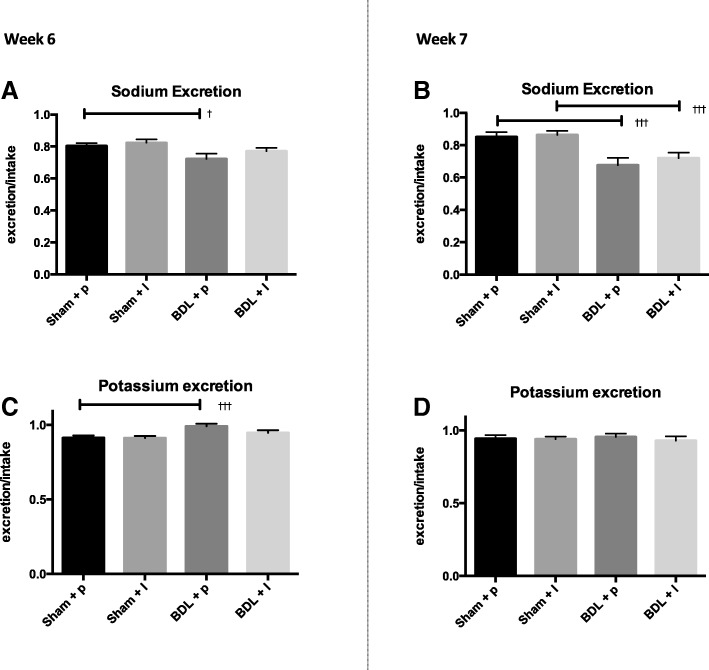
Urinary excretion of sodium and potassium for BDL groups and their respective controls after week 6 (left panel) and week 7 (right panel). Sodium and potassium excretion is calculated as excretion/intake. Values are expressed as mean ± SEM, ^†^*p* < 0.05: Sham vs. BDL, ^††^*p* < 0.01 Sham vs. BDL, ^†††^*p* < 0.001: Sham vs. BDL

During week 6 after surgery the renal sodium excretion was decreased in the BDL animals (sham+ placebo: 0.80 ± 0.02 vs. BDL+ placebo: 0.72 ± 0.03, *p* < 0.05, Fig. 2a). Losartan treatment did not affect the sodium excretion in the BDL animals compared to placebo (BDL + losartan: 0.77 ± 0.02, *p* > 0.05, Fig. 2a). At week 7 after BDL the renal sodium excretion was even lower in the BDL (sham + placebo: 0.85 ± 0.03 vs. BDL + placebo: 0.68 ± 0.04, *p* < 0.001, Fig. 2b). This was not affected by losartan treatment (BDL + losartan: 0.72 ± 0.03 *p* > 0.05, Fig. 2b). During week 6 after BDL the renal potassium excretion increased in the BDL animals (sham + placebo: 0.91 ± 0.02 vs. BDL + placebo: 0.99 ± 0.02, *p* < 0.001, Fig. 2c). Losartan treatment tended to decrease the potassium excretion and it was similar to the sham animals (sham + losartan 0.91 ± 0.01 vs. BDL losartan 0.95 ± 0.02, *p* > 0.05, Fig. 2c). During week 7 the urinary potassium excretion was similar in all four groups (Fig. 2d).

### Neurohumoral activity and hemodynamics

**Fig. 3:**
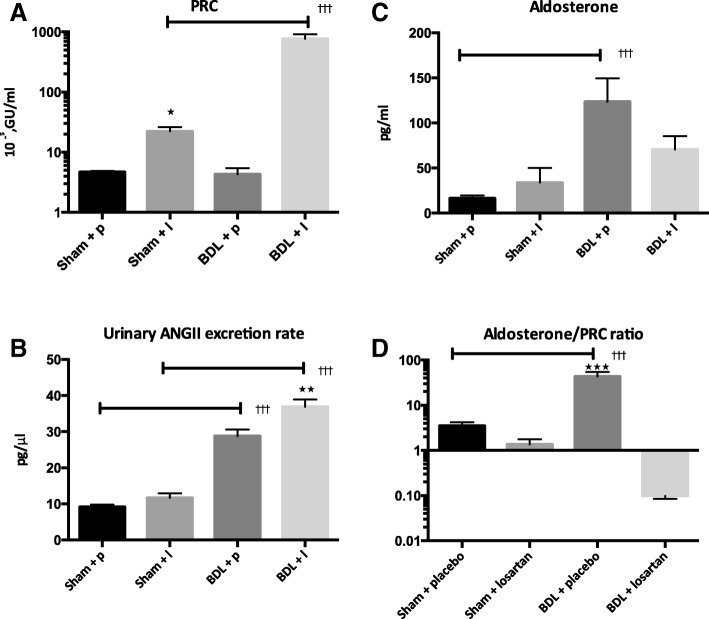
Renin and aldosterone concentration in plasma after 2 weeks of treatment. Urinary excretion rate of ANGII and the aldosterone/PRC ratio. Values are expressed as mean ± SEM. ***p < 0.001: BDL placebo vs. BDL losartan, ^†††^*p* < 0.001: Sham vs. BDL

The PRC was similar in the two placebo groups (sham: 4.7 ± 0.2 vs. BDL: 4.3 ± 1.1 10^−5^GU/ml, p > 0.05, Fig. 3a). Treatment with losartan increased PRC markedly (BDL + losartan: 769.3 ± 143.7 10^−5^GU/ml, *p* < 0.001, Fig. 3a). The urinary excretion of ANG II was increased in placebo treated BDL group compared to the sham control (sham+ placebo: 9.8 ± 0.4 vs. BDL+ placebo: 24.9 ± 1.0 pg/μl, *p* < 0.001). Losartan treatment increased ANGII excretion further in the BDL animals compared to the placebo treated BDL group (BDL + losartan: 35.4 ± 1.5 pg/μl, *p* < 0.01, Fig. 3b). BDL induced an increase in P-Aldosterone as expected (sham+ placebo: 16.4 ± 3.0 vs. BDL + placebo vs. BDL+ placebo: 123 ± 25.7 pg/ml, *p* < 0.001, Fig. 3c). Although ANGII excretion was increased in the losartan treated animals there was a trend of a lower P-Aldosterone in these animals compared to the placebo treated BDL animals (Fig. 3c). Considering the aldosterone/PRC ratio the aldosterone response to renin stimulus losartan treatment of the BDL animals markedly reduced the ratio (BDL + losartan: 0.01 ± 0.03 vs. BDL+ placebo: 43.5 ± 31.7, *p* < 0.001, Fig. 3d).

Invasive blood pressure was measured in two BDL operated animals, one placebo treated and one losartan treated. After onset of treatment with losartan there was a decrease in blood pressure of approximately 25% compared to baseline in the losartan treated rat whereas the blood pressure remained practically unchanged in the placebo treated BDL rat. BDL + losartan: 95.7 ± 23.2 to 74.1 ± 7.0 mmHg vs. BDL+ placebo: 114.8 ± 18.6 to 111.7 ± 4.7 mmHg (week 1 vs. week 7).

## Discussion

The treatment with losartan 4 mg/kg did not improve sodium excretion in the BDL rat model of cirrhosis in the late course of disease. As the smaller spleen size reflects reduced portal hypertension, and fewer animals had ascites in the losartan treated animals, some effect of losartan may have been present. The otherwise normal PRC in BDL rats increased 150 fold after losartan, which may be due to losartan-induced hypotension. This is supported by the 25% decrease in blood pressure in a single rat after losartan. We demonstrated elevated urinary ANGII levels reflecting an importance of intrarenal RAS in the BDL rat, which was further increased after losartan. The chosen dose of losartan blocked the AT-1 receptor efficiently as demonstrated by the trend towards reduced p-Aldosterone levels and reduced PRC/Aldosterone ratio. The renal sodium retention in the BDL animals was evident at week 6 and 7 after surgery. This is coherent with the fact that the animals develop ascites in the late phase of the disease [10]. Apart from the possible hemodynamic effect of losartan, the increase of PRC in the sham+losartan group might reflect the loss of angiotensin type 1 receptor mediated negative feedback on renin secretion by losartan. It is supported by the fact that renal angiotensin levels in BDL + losartan were higher than in BDL + placebo.

The increased potassium excretion in the BDL animals during the 6th week after surgery is explained by increased P-Aldosterone in the diseased animals. This was not found in the terminal phase of the disease (week 7). However as we did not measure fecal electrolyte excretions we are not able to calculate the precise balances of sodium and potassium. We have previously shown that BDL rats have significantly higher intestinal potassium and sodium excretions as compared to their controls at the end of week 7 and similar renal potassium excretions [11]. The fecal sodium and potassium excretions at the end of week 6 have not previously been studied.

In a previous study enhanced sodium excretion was found when losartan was given at dose of 0.5 mg/kg/day for one week starting at week 5 after surgery, whereas 10 mg/kg/day lead to deterioration of renal function and more pronounced sodium retention than seen in the control animals [9]. In a second study, long term treatment of losartan 5 and 10 mg/kg/day was given during early course of disease for 4 weeks in BDL model, and for 9 weeks the CCl_4_ model. Both doses were well tolerated in the CCl_4_ model but induced significantly reduced blood pressure in the BDL model. None of the doses improved sodium excretion [8]. In a preliminary study in the BDL model, 10 mg/kg/day induced a marked decrease in blood pressure, whereas a dose of 6 mg/kg day for 1 week at week 3 after surgery was well tolerated and reduced portal pressure [14]. The acute response to losartan infusion decreased MAP significantly at the doses 10 mg/kg and 30 mg/kg but not 3 mg/kg at week 5 [14]. In this study a dose of 4 mg/kg divided in two was anticipated to be sufficient to block the ANGII effect in the kidneys and at the same time be tolerated. Yet it seemed that the dosage of 4 mg/kg of losartan in the present study was poorly tolerated in the BDL rats. Judging from the PRC response in the BDL-losartan group this hypotensive effect was pronounced. The suggested hypotensive effect of the dosage used was also evident in the blood pressure measurement in a single rat in which it was reduced by 25%. However blood pressure measurement in only one animal is not sufficient to support this conclusion. Other indicators of profound side effects of the losartan treatment were found in the BDL rats. The mortality was higher and the P-Potassium was higher. Summing up these effects, there is an indication of a pronounced hemodynamic impairment in this animal model of cirrhosis [15]. Although this proposed mechanism and role of hemodynamic deterioration is based on incomplete data we find it plausible.

Intolerability to losartan in the BDL model could be explained by the biliary elimination of losartan of 50–60% [16], where the metabolite is pharmacologically more active. Severe cholestasis develops in the BDL model, and accumulation of losartan metabolites may be partly responsible for deleterious hemodynamic and succeeding renal deterioration [17].

In a series of human experiment the role of ANGII antagonist in treatment of sodium retention has been explored. In a dose-response study renal sodium retention was recovered by 7.5 mg losartan in patients with preascitic cirrhosis. A decrease in mean arterial pressure was observed when treated with 10 mg losartan [18]; and the sodium retention induced by erect posture was blunted by 7.5 mg of losartan [19]. Patients treated with transjugular intrahepatic portosystemic shunt (TIPS), reducing or normalizing portal pressure [20], still show impaired sodium handling similar to that of patients with pre-ascitic cirrhosis [21]. Treatment with losartan 7.5 mg given to patients treated with TIPS improved sodium excretion during upright position [22]. The effects of the low dose of losartan were attributed to the intrarenal component of ANGII. A short term dose of 25 mg of losartan given to compensated and decompensated cirrhotic patients has been shown to improve natriuresis without renal impairment [23]. We measured urinary ANG II as an indicator of intrarenal RAAS activation. We demonstrated that despite normal PRC, the placebo treated rat BDL rat has elevated urinary ANG II level indicating increased intrarenal RAAS activation, which support a role of intrarenal RAAS activation in cirrhosis. The role of ANGII receptor antagonists in the treatment of sodium retention is not yet established. Emerging evidence suggests ANGII receptor antagonists as effective treatment of both portal hypertension as well as liver fibrosis, thus the deleterious renal effect is of concern [24, 25].

## Conclusions

Losartan 4 mg/kg/day did not increase sodium excretion in this model of liver cirrhosis although the intrarenal ANGII was increased. However losartan reduced spleen size, and the presence of ascites. Losartan induced increased urinary excretion of ANGII and trend towards reduced aldosterone response indicating sufficient blockage of the receptor. However, losartan was not well tolerated in the BDL animals, and the expected effect on sodium excretion was not seen, on the contrary, a 150-fold increase in PRC was induced. The effect of an ANGII antagonist has not previously been studied in the late phase of the disease and there fore this study provided important information about the potential deleterious effects of the treatment. Lower doses of losartan in the same model would have explored the possibility of natriuretic effects in this stage of disease.

## Abbreviations

ANGII: Angiotensin II
BDL: Bile duct ligation.
PRC: Plasma renin concentration.
RAAS: Renin angiotensin aldosterone systeme.

## References

[1] Sola E, Gines P (2010). Renal and circulatory dysfunction in cirrhosis: current management and future perspectives. J Hepatol.

[2] Lumbers ER (1999). Angiotensin and aldosterone. Regul Pept.

[3] Lavoie JL, Sigmund CD (2003). Minireview: overview of the renin-angiotensin system--an endocrine and paracrine system. Endocrinology.

[4] Martinez-Prieto C, Ortiz MC, Fortepiani LA, Ruiz-Macia J, Atucha NM, Garcia-Estan J (2000). Haemodynamic and renal evolution of the bile duct-ligated rat. Clin Sci (Lond)..

[5] Thiesson HC, Jensen BL, Bistrup C, Ottosen PD, McNeilly AD, Andrew R, Seckl J, Skott O (2007). Renal sodium retention in cirrhotic rats depends on glucocorticoid-mediated activation of mineralocorticoid receptor due to decreased renal 11beta-HSD-2 activity. Am J Physiol Regul Integr Comp Physiol.

[6] Yang YY, Lin HC, Huang YT, Lee TY, Hou MC, Lee FY, Liu RS, Chang FY, Lee SD (2002). Effect of 1-week losartan administration on bile duct-ligated cirrhotic rats with portal hypertension. J Hepatol.

[7] Ubeda M, Matzilevich MM, Atucha NM, Garcia-Estan J, Quesada T, Tang SS, Ingelfinger JR (1994). Renin and angiotensinogen mRNA expression in the kidneys of rats subjected to long-term bile duct ligation. Hepatology.

[8] Croquet V, Moal F, Veal N, Wang J, Oberti F, Roux J, Vuillemin E, Gallois Y, Douay O, Chappard D (2002). Hemodynamic and antifibrotic effects of losartan in rats with liver fibrosis and/or portal hypertension. J Hepatol.

[9] Heller J, Trebicka J, Shiozawa T, Schepke M, Neef M, Hennenberg M, Sauerbruch T (2005). Vascular, hemodynamic and renal effects of low-dose losartan in rats with secondary biliary cirrhosis. Liver Int.

[10] Kountouras J, Billing BH, Scheuer PJ (1984). Prolonged bile duct obstruction: a new experimental model for cirrhosis in the rat. Br J Exp Pathol.

[11] Accatino L, Contreras A, Berdichevsky E, Quintana C (1981). The effect of complete biliary obstruction on bile secretion. Studies on the mechanisms of postcholestatic choleresis in the rat. J Lab Clin Med.

[12] Poulsen K, Jorgensen J (1974). An easy radioimmunological microassay of renin activity, concentration and substrate in human and animal plasma and tissues based on angiotensin I trapping by antibody. J Clin Endocrinol Metab.

[13] Bie P, Sandgaard NC (2000). Determinants of the natriuresis after acute, slow sodium loading in conscious dogs. Am J Physiol Regul Integr Comp Physiol..

[14] Heller J, Shiozawa T, Trebicka J, Hennenberg M, Schepke M, Neef M, Sauerbruch T (2003). Acute haemodynamic effects of losartan in anaesthetized cirrhotic rats. Eur J Clin Investig.

[15] Cervenka L, Wang CT, Navar LG (1998). Effects of acute AT1 receptor blockade by candesartan on arterial pressure and renal function in rats. Am J Phys.

[16] Christ DD, Wong PC, Wong YN, Hart SD, Quon CY, Lam GN (1994). The pharmacokinetics and pharmacodynamics of the angiotensin II receptor antagonist losartan potassium (DuP 753/MK 954) in the dog. J Pharmacol Exp Ther.

[17] Bomzon A, Rosenberg M, Gali D, Binah O, Mordechovitz D, Better OS, Greig PD, Blendis LM (1986). Systemic hypotension and decreased pressor response in dogs with chronic bile duct ligation. Hepatology.

[18] Girgrah N, Liu P, Collier J, Blendis L, Wong F (2000). Haemodynamic, renal sodium handling, and neurohormonal effects of acute administration of low dose losartan, an angiotensin II receptor antagonist, in preascitic cirrhosis. Gut.

[19] Wong F, Liu P, Blendis L (2002). The mechanism of improved sodium homeostasis of low-dose losartan in preascitic cirrhosis. Hepatology.

[20] Skeens J, Semba C, Dake M (1995). Transjugular intrahepatic portosystemic shunts. Annu Rev Med.

[21] Wong W, Liu P, Blendis L, Wong F (1999). Long-term renal sodium handling in patients with cirrhosis treated with transjugular intrahepatic portosystemic shunts for refractory ascites. Am J Med.

[22] Therapondos G, Hol L, Benjaminov F, Wong F (2006). The effect of single oral low-dose losartan on posture-related sodium handling in post-TIPS ascites-free cirrhosis. Hepatology.

[23] Yang YY, Lin HC, Lee WC, Hou MC, Lee FY, Chang FY, Lee SD (2002). One-week losartan administration increases sodium excretion in cirrhotic patients with and without ascites. J Gastroenterol.

[24] Grace JA, Herath CB, Mak KY, Burrell LM, Angus PW (2012). Update on new aspects of the renin-angiotensin system in liver disease: clinical implications and new therapeutic options. Clin Sci (Lond).

[25] Tandon P, Abraldes JG, Berzigotti A, Garcia-Pagan JC, Bosch J (2010). Renin-angiotensin-aldosterone inhibitors in the reduction of portal pressure: a systematic review and meta-analysis. J Hepatol.

